# Interplay Between Oxygen-Dependent Marangoni Convection and Recoil Pressure in Laser Powder Bed Fusion of Ti-15Zr Alloys

**DOI:** 10.3390/ma19214653

**Published:** 2026-10-31

**Authors:** Xikai Zhou, Hongliang Xiang, Qinchang Wu, Jiawei Liu, Xiaoqiang Cai, Gang Mu, Yang Li, Chaochao Wu

**Affiliations:** 1 School of Mechanical Engineering and Automation, Fuzhou University, Fuzhou 350108, China; 2 School of Advanced Manufacturing, Fuzhou University, Jinjiang 362251, China; 3 School of Materials Science and Engineering, Nanjing University of Science and Technology, Nanjing 210094, China; 4 School of Mechanical Engineering, Tsinghua University, Beijing 100084, China

**Keywords:** laser powder bed fusion, oxygen content, Marangoni convection, multi-physics modeling, molten pool dynamics, recoil pressure

## Abstract

Ti-15Zr alloys offer favorable biocompatibility, and interstitial oxygen provides an attractive pathway for further strengthening. However, the interaction between oxygen-modified Marangoni convection and evaporation-induced recoil pressure during laser powder bed fusion (LPBF) remains insufficiently understood. A three-dimensional multiphysics thermal-fluid model incorporating recoil pressure and temperature- and oxygen-dependent surface tension was developed. Surface-tension-only, recoil-pressure-only, and coupled simulations were compared on an idealized solid substrate, followed by discrete powder-bed simulations. Increasing oxygen content shifts the dominant Marangoni flow from outward spreading to inward convergence. Recoil pressure primarily drives central depression, whereas Marangoni convection redistributes molten metal. In transverse sections, low-oxygen outward flow competes with recoil-driven downward transport and promotes lateral spreading, whereas high-oxygen inward flow reinforces downward circulation. In longitudinal sections, reversed Marangoni flow suppresses recoil-driven rearward transport and promotes recirculation behind the vapor depression. Increasing oxygen content from 0 to 1.0 wt.% raises the top-view projected-area fraction with *dσ*/*dT* > 0 from 41.8% to 63.0%. Under powder-bed conditions, the mean track width in the central region decreases from 61.32 to 51.23 μm (16.5%), while the mean depth increases from 47.71 to 49.95 μm (4.7%). The findings provide a physical basis for regulating melt pool evolution in LPBF Ti-15Zr alloys.

## 1. Introduction

Ti-15Zr alloys have attracted increasing interest for biomedical applications because of their favorable biocompatibility, corrosion resistance, and mechanical performance. In particular, interstitial oxygen offers an effective route for further strengthening Ti-Zr alloys without introducing potentially toxic alloying elements, providing additional flexibility for tailoring their performance through composition control [1]. Laser Powder Bed Fusion (LPBF), with its capability for near-net-shape fabrication of geometrically complex metallic components [2,3], has been widely applied in aerospace, biomedical, and advanced engineering fields [2]. This also provides a promising manufacturing route for such Ti-Zr-based alloys [4].

However, oxygen is not merely a strengthening element of built components. During the melting process, as a surface-active element, variations in oxygen content can substantially modify the temperature dependence of surface tension and, consequently, the magnitude and even direction of thermocapillary Marangoni convection. Such oxygen-induced changes in melt flow may strongly influence molten-metal redistribution, melt pool morphology, and the final solidified track. With the advanced experimental techniques like in situ synchrotron X-ray imaging [5], Yang et al. [6] quantitatively mapped the velocity field in the 316L stainless steel melt pool using tungsten tracer particles and found that when oxygen concentration exceeds a critical threshold, inward Marangoni convection is triggered, significantly reducing the laser power density required for the transition from conduction mode to keyhole mode. This indicates that the influence of oxygen becomes particularly complex under LPBF conditions because Marangoni convection does not act independently.

Marangoni convection and recoil pressure due to evaporation [7,8] constitute two major interfacial driving mechanisms governing melt pool dynamics, but they redistribute molten metal through fundamentally different pathways. The recoil pressure drives strong downward melt flow, increasing penetration depth while reducing keyhole stability, thereby inducing irregular oscillations and pore formation [9,10]. The transition from conduction mode to keyhole mode is essentially governed by the dynamic balance among recoil pressure, surface tension, and hydrostatic pressure [11]. Zhao et al. [12] conducted systematic parametric studies and showed that the conduction-to-keyhole transition threshold is not determined solely by laser power density, but is also strongly regulated by Marangoni flow direction.

Resolving these coupled multi-physics phenomena is of great importance for better understanding and controlling of the printing process, where numerical models are powerful tools. Over the past decade, substantial progress has been particularly made in high-fidelity computational modeling of melt pool behavior in LPBF. Khairallah et al. [13] established a fundamental understanding of melt flow physics through high-fidelity powder-scale simulations, and demonstrated that recoil pressure and Marangoni convection are the dominant driving forces. More recently, Yu et al. [8] further developed an integrated numerical framework by incorporating metal vapor condensation dynamics, quantitatively characterizing key factors controlling keyhole pore evolution, including vapor condensation, liquid vortex formation, recoil pressure, surface tension, and keyhole morphology. These studies clarified the critical evolution stages of keyhole porosity, from keyhole formation to pore collapse, bifurcation, and solidification-induced entrapment. Vaglio et al. [14] proposed a novel thermal-geometric model for keyhole pore prediction by integrating vaporization-driven recoil pressure and temperature-dependent thermophysical properties. Collectively, these studies have established the foundation for high-fidelity LPBF modeling by progressively incorporating increasingly realistic descriptions of multiphysics interactions [15]. Beyond keyhole porosity, high-fidelity simulations of electron beam powder bed fusion have also linked powder-scale melt flow to track irregularity and surface morphology [16,17].

Regarding the influence of oxygen content on melt flow, numerical simulations further showed that with increasing oxygen concentration, the surface tension gradient coefficient (*dσ*/*dT*) changes from negative to positive, thereby reversing Marangoni flow direction [18,19]. Chia et al. [20] incorporated oxygen transport and oxygen-dependent surface tension into a multiphysics thermal-fluid model of LPBF. Their results showed that oxygen from the powder, substrate, and atmosphere can substantially alter Marangoni circulation and oxygen redistribution, even when changes in overall melt pool dimensions remain limited under the investigated conditions. They further attributed oxygen-rich surface streaks to recoil-pressure-induced fluid accumulation and rapid solidification at the track edges, demonstrating that oxygen redistribution cannot be explained by Marangoni convection alone.

Nevertheless, investigations on the melt pool dynamics for Ti-15Zr alloy with oxygen addition under LPBF conditions remain limited. Particularly, changes in oxygen content can reverse the direction of Marangoni convection, thereby altering whether the thermocapillary flow competes with or reinforces the recoil-pressure-driven flow. Despite its importance, the interaction between oxygen-dependent Marangoni convection and evaporation-induced recoil pressure, and how their coupling governs melt pool evolution in LPBF Ti-15Zr alloys, remains insufficiently understood. Our previous study [4] examined the mechanical and wear properties of oxygen-modified Ti-15Zr. The present work examines how oxygen-dependent surface tension interacts with recoil pressure to modify melt circulation and single-track geometry under solid-substrate and powder-bed conditions.

To bridge these knowledge gaps, this study develops a high-fidelity thermo-fluid coupled model incorporating temperature-dependent thermophysical properties, oxygen-dependent surface tension, and evaporation-induced recoil pressure to investigate the oxygen-dependent interplay between Marangoni convection and recoil pressure during melt pool evolution. Particular attention is devoted to elucidating how oxygen regulates the transition of Marangoni flow mode and how the interplay between Marangoni convection and recoil pressure governs melt pool formation mechanisms. Furthermore, the effects of oxygen-induced melt flow evolution on melt track geometry are quantitatively evaluated. This study establishes the mechanistic relationship linking oxygen content, surface tension gradient, melt flow transition, and melt pool evolution, thereby providing theoretical guidance for oxygen-controlled alloy design and process optimization in LPBF.

## 2. Experiments and Methods

### 2.1. Powder Feedstock and Single-Track Experiments

The Ti-15Zr alloy powder and TiO_2_ nanoparticles were blended using a planetary high-energy ball mill (QM-3SP04, Nanjing, China) under vacuum conditions. TiO_2_ nanoparticles were used as the oxygen source, and powder mixtures with different nominal oxygen contents were prepared by varying the TiO_2_ addition. Ball milling was used to promote the dispersion of TiO_2_ nanoparticles on the alloy powder surfaces. Detailed powder characterization was reported in our previous study [4].

Single-track experiments were conducted using a commercial LPBF system (e-plus-m150, Beijing, China) equipped with a 1064 nm fiber laser with a maximum power of 500 W and a beam radius of 90 μm. The experiments were performed under an argon atmosphere with a residual oxygen content of approximately 0.1%. Laser powers of 100, 200, 300, and 400 W were used at a constant scanning speed of 1.0 m/s. The average track width was evaluated in the central steady-state region of each track to reduce the influence of laser start-stop transients and boundary effects. These measurements were used to validate the simulated single-track geometry. The elemental distributions of the as-built Ti-15Zr-0.2O and Ti-15Zr-0.4O samples were characterized using a field-emission electron probe microanalyzer (EPMA, JXA-iHP200F, JEOL Ltd., Tokyo, Japan).

### 2.2. Multiphysics Model

A numerical framework was developed to examine the interaction between oxygen-dependent Marangoni convection and evaporation recoil pressure during LPBF of Ti-15Zr-*x*O alloys. The framework combined three components with distinct roles: the discrete element method (DEM) generated the initial powder-bed geometry, computational fluid dynamics (CFD) resolved transient heat transfer, fluid flow, and free-surface evolution, and the discrete phase model (DPM) described the transport and thermal response of dispersed TiO_2_ particles in the solid-substrate simulations. The driving mechanisms were first examined under an idealized substrate condition, before their combined effects were evaluated in a discrete powder bed.

#### 2.2.1. Initial Powder-Bed Reconstruction

The initial packing of the alloy powder was reconstructed using DEM. Each powder particle was treated as a discrete body with translational and rotational degrees of freedom. Contact interactions were calculated from particle overlap and relative motion, with the normal and tangential components evaluated using Equations (1) and (2), respectively [16,21].

(1) $$\begin{array}{c} \vec{F_{n}}=-\max(0,\;k_{n}\delta_{\mathrm{ij}}+c_{n}\frac{d}{\mathrm{dt}}\delta_{\mathrm{ij}})\vec{e_{n}} \end{array}$$

(2) $$\begin{array}{c} \vec{F_{t}}=-\min(c_{t}u_{t},\;\mu_{f}F_{n})\vec{e_{t}} \end{array}$$

Here, *k_n_* is the normal spring stiffness, *δ_ij_* is the overlap between contacting particles, and *c_n_* and *c_t_* are the normal and tangential damping coefficients. The quantities *μ_f_* and *u_t_* denote the dynamic friction coefficient and the tangential relative speed, respectively, while *e_n_* and *e_t_* are the corresponding unit vectors. Particle translation and rotation were obtained from the resultant force and torque:

(3) $$\begin{array}{c} m_{i}\frac{d^{2}\vec{x_{i}}}{dt^{2}}=\sum_{i}\vec{F_{i}} \end{array}$$

(4) $$\begin{array}{c} I_{i}\frac{d\vec{\omega_{i}}}{\mathrm{dt}}=\sum_{i}\vec{T_{i}} \end{array}$$

In Equations (3) and (4), *m_i_* and *I_i_* are the mass and moment of inertia of particle *i*, *x_i_* is its position, and *ω_i_* is its angular velocity. *F_i_* and *T_i_* are the resultant force and torque, respectively. The resulting packing geometry was used to initialize the metal and gas regions in the CFD domain. Thus, DEM supplied the initial powder-bed configuration rather than describing particle contacts during laser melting.

#### 2.2.2. Melt Pool Thermo-Fluid Model

The CFD calculation resolved three-dimensional heat transfer and fluid flow during laser-induced melting and subsequent solidification. The model included laser heating, viscous flow, gravity, capillary and Marangoni forces, evaporation recoil pressure, and evaporative heat loss. The metallic phase was represented by Ti-15Zr, and the analysis focused on oxygen effects while neglecting contributions from other trace elements. The governing equations were discretized using the finite volume method. Conservation of mass, momentum, and energy was expressed as follows [22,23,24]:

(5) $$\begin{array}{c} \frac{\partial \rho }{\partial t}+\nabla \cdot \left(\rho \vec{v}\right)=0 \end{array}$$

(6) $$\begin{array}{c} \frac{\partial \left(\rho \vec{v}\right)}{\partial t}+\nabla \cdot \left(\rho \vec{v}⊗\vec{v}\right)=-\nabla p+\nabla \cdot \left(\mu \nabla \vec{v}\right)+\rho \vec{g}-{\vec{F}}_{D}+{\vec{F}}_{\mathrm{st}}+{\vec{F}}_{\mathrm{recoil}}+{\vec{F}}_{\mathrm{sp}} \end{array}$$

(7) $$\begin{array}{c} \frac{\partial \mathrm{\rho h}}{\partial t}+\nabla \cdot \left(\rho \vec{v}h\right)=\nabla \cdot \left(k\nabla T\right)+Q_{\mathrm{laser}}-Q_{\mathrm{evap}}+Q_{p} \end{array}$$

Here, *ρ*, $\vec{v}$, *p*, and *μ* denote the local density, fluid velocity, pressure, and dynamic viscosity, respectively; *g* is gravitational acceleration; and *h*, *k*, and *T* are specific enthalpy, thermal conductivity, and temperature. The momentum sources represent solidification-induced resistance ${\vec{F}}_{D}$, surface-tension forces ${\vec{F}}_{\mathrm{st}}$, evaporation recoil ${\vec{F}}_{\mathrm{recoil}}$, and particle-fluid momentum exchange ${\vec{F}}_{\mathrm{sp}}$. In the energy equation, *Q_Laser_* and *Q_evap_* represent laser heating and evaporative heat loss, respectively, while *Q_p_* accounts for heat exchange with the tracked particles.

Flow resistance in the partially solidified metal was represented by a liquid-fraction-dependent damping term:

(8) $$\begin{array}{c} {\vec{F}}_{D}=A_{\mathrm{mush}}\left[\frac{{\left(1-f_{l}\right)}^{2}}{f_{l}^{3}+c_{k}}\right]\vec{v} \end{array}$$

In Equation (8), *f_l_* is the liquid fraction within the metallic phase, *A_mush_* controls the strength of momentum damping, and *c_k_* is a small positive dimensionless constant that prevents a zero denominator. This term suppresses fluid motion as the metal solidifies.

The surface tension term ${\vec{F}}_{\mathrm{st}}$ was modeled using the Continuous Surface Force model (CSF) and transformed into a volumetric source term in the momentum equation.

(9) $$\begin{array}{c} {\vec{F}}_{\mathrm{st}}=2\mathrm{\sigma k\rho}\nabla \alpha \frac{2\rho }{\rho_{s}+\rho_{g}} \end{array}$$

In this formulation, *σ* is the surface tension coefficient, *k* denotes interface curvature, and *ρ_s_* and *ρ_g_* are the densities of the metal and gas phases, respectively. It should be emphasized that the present model considered not only the normal capillary force governed by interface curvature but also explicitly incorporated the tangential shear stress induced by the surface tension gradient, namely the Marangoni term ((dσ/dT)∇T). This term directly determines the migration direction of the surface fluid and forms the physical basis for the oxygen-induced reversal of flow topology discussed in this work.

The evaporation recoil pressure ${\vec{F}}_{\mathrm{recoil}}$ was converted into a volumetric body force using the following formulation [25].

(10) $$\begin{array}{c} {\vec{F}}_{\mathrm{recoil}}=0.54p_{0}\exp\left[\frac{L_{v}\left(T-T_{v}\right)}{\mathrm{RT}T_{v}}\right]\nabla \alpha \frac{2\rho }{\rho_{s}+\rho_{g}} \end{array}$$

Here, *P*_0_ is the ambient pressure, *L_v_* is the latent heat of vaporization, *R* is the ideal gas constant, and *T_v_* corresponds to the boiling temperature of the metal. Laser heating was prescribed as a moving Gaussian surface heat flux with a fixed effective absorptivity of *η* = 0.6, as given in Equation (11). The absorbed heat input was applied at the gas–metal interface. Here, *P* is the incident laser power, *r* is the beam-radius parameter in Equation (11), (*x*_0_ *+ vt, y*_0_) is the instantaneous beam-center position, *v* is the scanning speed, and *t* is time.

(11) $$\begin{array}{c} Q_{\mathrm{Laser}}=\frac{3\mathrm{\eta P}}{\pi r^{2}}\exp\left[-3\frac{(x-x_{0}-\mathrm{vt}{)}^{2}+(y-y_{0}{)}^{2}}{r^{2}}\right] \end{array}$$

The evaporative heat loss *Q_evap_* was applied as a surface heat sink acting on the evaporation surface.

(12) $$\begin{array}{c} Q_{\mathrm{evap}}=0.82\frac{L_{v}}{\sqrt{2\mathrm{\pi MRT}}}p_{0}\exp\left[\frac{L_{v}\left(T-T_{v}\right)}{\mathrm{RT}T_{v}}\right] \end{array}$$

The VOF method was employed to update the free surface at each time step, as expressed in Equation (13) [26].

(13) $$\begin{array}{c} \frac{\partial F}{\partial t}+\left(\vec{u}\cdot \nabla \right)F=0 \end{array}$$

#### 2.2.3. Particle Transport and Thermal Response

DPM was used to examine the motion and heating of dispersed TiO_2_ particles in the evolving melt pool. The particles were tracked individually in a Lagrangian framework, with interparticle collisions neglected under the assumed dilute and well-dispersed conditions. Their momentum and thermal responses were described by:

(14) $$\begin{array}{c} \rho_{p}V_{p}\frac{{\vec{\mathrm{dv}}}_{p}}{\mathrm{dt}}={\vec{F}}_{d}+{\vec{F}}_{g}+{\vec{F}}_{p}+{\vec{F}}_{b} \end{array}$$

(15) $$\begin{array}{c} \rho_{p}V_{p}c_{p}\frac{dT_{p}}{\mathrm{dt}}=h_{p}A_{p}\left(T_{f}-T_{p}\right) \end{array}$$

In Equation (14), *ρ_p_* and *V_p_* denote particle density and volume, respectively, and ${\vec{v}}_{p}$ is particle velocity. The forces acting on a particle comprise drag ${\vec{F}}_{d}$, gravity ${\vec{F}}_{g}$, the pressure-gradient contribution ${\vec{F}}_{p}$, and buoyancy ${\vec{F}}_{b}$. Drag force is dominant and controls particle transport under Marangoni-driven flow. In Equation (15), *c_p_* and *T_p_* are the particle specific heat and temperature, *h_p_* is the particle-fluid heat-transfer coefficient, and *A_p_* is the particle surface area. The fluid temperature *T_f_* is evaluated at the instantaneous particle position, allowing the thermal response to follow particle motion through the nonuniform temperature field.

Two-way coupling was employed in the particle-tracking simulations. Particle-fluid momentum and heat exchange were volume-averaged and introduced into the continuous-phase equations through ${\vec{F}}_{\mathrm{sp}}$ and *Q_p_*, respectively. The resulting trajectories and thermal histories were used to examine particle transport and heating within the melt pool.

### 2.3. Simulation Setup

The computational domain measured 800 μm × 200 μm × 250 μm and was discretized using a structured hexahedral mesh with a cell size of 5 μm. The mesh sensitivity analysis is presented in Section 3.1. The initial temperature throughout the domain was set to 300 K. The oxygen-dependent simulations were performed at a laser power of 230 W and a scanning speed of 1.25 m/s. Laser heating was modeled as a moving Gaussian beam coupled with single-reflection ray tracing on the evolving melt-pool free surface, assuming a fixed effective absorptivity of 0.6 without multiple reflections.

Two geometric configurations were considered: an idealized solid substrate and a discrete powder bed reconstructed using DEM. The solid-substrate model was used to examine melt flow under three force configurations: surface tension only, recoil pressure only, and their coupled action. The surface-tension contribution included both normal capillary forces and tangential Marangoni stresses. Both surface-tension and recoil-pressure forcing were included in the powder-bed simulations to examine melt pool evolution and track formation in the presence of discrete powder geometry.

The prescribed oxygen contents were 0, 0.2, 0.4, and 1.0 wt.%, expressed as oxygen mass percentages. The 0.2 and 0.4 wt.% O simulations correspond to the nominal experimental compositions, with measured values of 0.19 and 0.41 wt.%. The 0 wt.% condition served as an idealized oxygen-free reference, while the 1.0 wt.% condition extended the analysis beyond the experimental composition range. For each composition, the dissolved oxygen content was prescribed as spatially uniform within the metallic phase. Its influence on interfacial flow was represented by the temperature- and composition-dependent surface tension, described in Section 3.3.

The thermophysical properties and model parameters are listed in Table 1. The same set of bulk matrix parameters was used for each prescribed oxygen content. The effects of dissolved oxygen and residual TiO_2_ on density, viscosity, and thermal conductivity were neglected, whereas oxygen-dependent surface tension was retained through $\sigma_{s}^{T}$.

In the model, the VOF method was used to distinguish the gas and metal phases, while the melting-solidification model distinguished the solid and liquid states within the metallic phase. Accordingly, the effective thermophysical property φ of any computational cell can be expressed as follows:

(16) $$\begin{array}{c} \bar{\varphi }=F\left(1-f_{l}\right)\varphi_{s}+Ff_{l}\varphi_{l}+\left(1-F\right)\varphi_{g} \end{array}$$

Here, *φ_s_*, *φ_l_*, and *φ_g_* represent the properties of the solid metal, liquid metal, and gas, respectively. The weighting factors *F*(1 − *f*_l_), *Ff*_l_, and (1 − *F*) specify their contributions to the effective cell property. This formulation was used for the phase-dependent density, thermal conductivity, and specific heat.

## 3. Results and Discussions

### 3.1. Validation and Mesh Sensitivity of the Melt Pool Model

Mesh sensitivity was assessed using three structured hexahedral meshes with cell sizes of 10, 5, and 2.5 μm, corresponding to 40,000, 320,000, and 2,560,000 cells, respectively. Relative to the 2.5 μm mesh, the mean track widths and depths differed by approximately 1.3–1.6% for the 5 μm mesh and 8.3–8.9% for the 10 μm mesh (Table 2). The medium mesh was therefore adopted for the subsequent simulations to balance numerical accuracy and computational cost.

**Figure 1:**
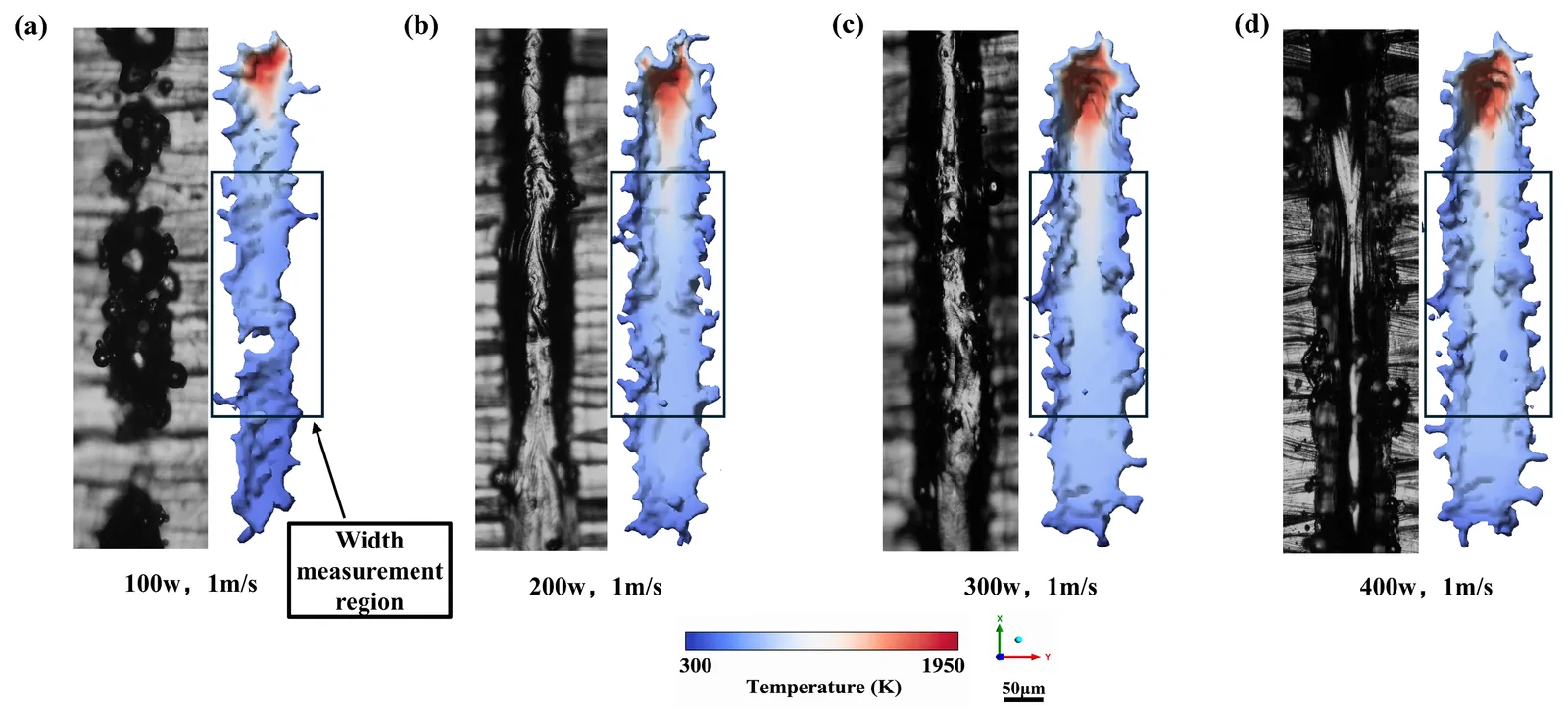
Comparison between experimental and simulated single-track morphologies under different laser powers: (**a**) 100 W; (**b**) 200 W; (**c**) 300 W; (**d**) 400 W (scan speed: 1 m/s).

The predictive capability of the developed model was first validated against experimental measurements. The simulated single-track widths were compared with experimental measurements at a constant scanning speed of 1 m/s and laser powers of 100, 200, 300, and 400 W. To minimize the influence of laser start-stop transients and boundary effects, only the steady-state region located at the middle of the melt track (marked by the black rectangle in Figure 1) was used to determine the average track width.

As summarized in Table 3, the experimental and simulated track widths both increase with laser power. Validation was limited to track width at laser powers of 100–400 W and a scanning speed of 1.0 m/s, with systematic overprediction of 5.6–7.5%. Although 230 W lies within the tested power range, the oxygen-dependent width and depth changes at 1.25 m/s have not been directly validated and are presented as model predictions.

### 3.2. Transportation and Thermal History of TiO_2_ Particles

Since oxygen release from TiO_2_ particles is the prerequisite for oxygen-induced surface tension variation investigated in the following sections, the transport and thermal response of TiO_2_ particles are then analyzed. Based on the validated model, the thermal history of TiO_2_ particles, particle transport and thermal exposure, and their coupled effects on the surface tension gradient, Marangoni convection, and melt track evolution are further investigated.

In this subsection, the powder-bed structure was not explicitly included; instead, the melt pool was treated as an equivalent continuum. This simplification was based on the fact that most powder particles participating in melt pool formation rapidly melted during LPBF, and the subsequent heat transfer and flow behavior were dominated by the liquid metal. Previous studies have shown that treating the powder bed as an equivalent continuum significantly reduces computational cost while preserving sufficient accuracy in describing melt pool evolution.

**Figure 2:**
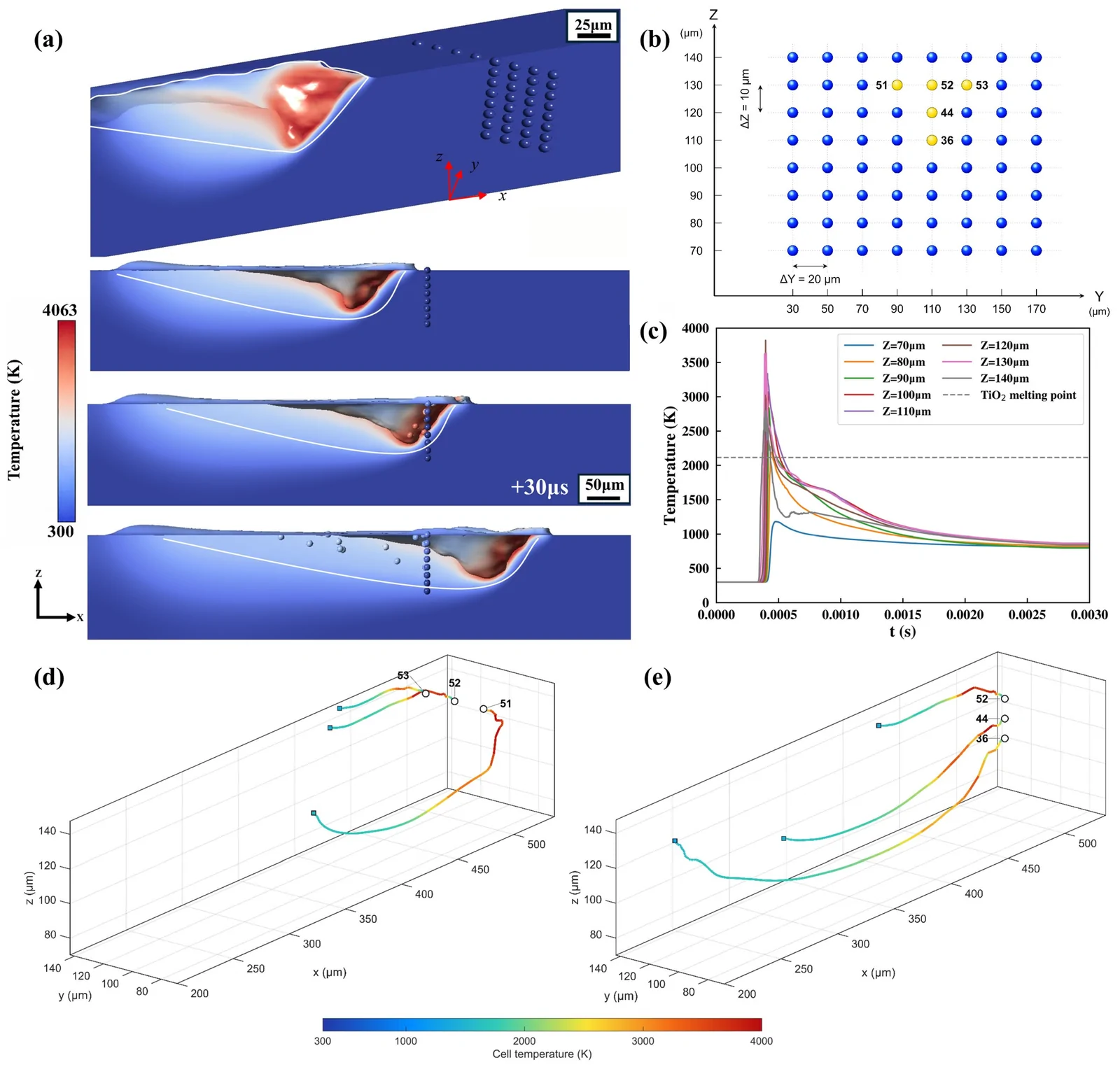
Transport and thermal histories of TiO_2_ tracer particles during LPBF (P = 230 W, v = 1.25 m/s): (**a**) melt pool evolution; (**b**) initial tracer arrangement (yellow: selected tracers in (**d**,**e**); blue: remaining tracers); (**c**) temperature histories of tracers initially arranged in the Y = 110 μm column; (**d**,**e**) three-dimensional trajectories of particles selected at different initial transverse positions and depths, respectively, colored by the local cell temperature at the corresponding time (open circles mark initial positions; filled squares mark recorded endpoints).

To investigate the transport behavior and thermal histories of TiO_2_ particles in the melt pool, tracers with a nominal diameter of 20 nm were initially embedded in the simulation domain, rather than reproducing the actual distribution of TiO_2_ nanoparticles on the Ti-15Zr powder surfaces. As shown in Figure 2b, the tracers were symmetrically arranged in an 8 × 8 matrix below the scan centerline to compare their thermal histories at different depths and lateral positions. As scanning proceeded, a quasi-steady melt pool developed, as shown in Figure 2a. The red region represents metal above the solidus temperature of Ti-15Zr (T_s_ = 1880 K). Considering the narrow melting range of Ti-15Zr (1880–1950 K), the phase transformation was simplified as occurring at a single solidus temperature to improve computational efficiency [31].

Under quasi-steady conditions, the melt pool dimensions were approximately 68 μm in width, and 45 μm in depth. The peak local temperature gradient on the free surface reached approximately 10^7^ K/m, which induced a strong surface tension gradient and consequently intense Marangoni convection, driving the molten metal radially outward from the laser interaction zone toward the melt pool boundary. These coupled thermal-fluid effects established the fundamental internal flow structure of the melt pool.

Figure 2a illustrates the evolution of tracer-particle positions and melt pool morphology during scanning. The initially ordered particle distribution is progressively distorted as the tracers are entrained in the molten region. Their displacement toward the trailing region is accompanied by redistribution in both the transverse and depth directions, indicating that particle transport follows the three-dimensional melt pool flow rather than simple translation along the scanning direction.

In addition, the melt pool depth in LPBF generally exceeds the thickness of a single powder layer, allowing subsequent laser passes to remelt the previously solidified region and further heat particles that were not fully melted during the initial scan. Consequently, the evolution of TiO_2_ particles should be considered as a thermal-history-dependent process of progressive melting and oxygen release. The thermal exposure of a particle depends on its trajectory through the transient melt pool. The internal thermal response of the tracers was therefore assessed using the characteristic thermal-diffusion time, estimated for a spherical particle as follows:

(17) $$\begin{array}{c} T_{\infty }\approx \frac{r^{2}}{\alpha } \end{array}$$

(18) $$\begin{array}{c} \alpha =k/\left(\rho \cdot C_{P}\right) \end{array}$$

where *r* is the particle radius and α is the thermal diffusivity. The key thermophysical parameters of TiO_2_ dioxide are as follows: *ρ* ≈ 4260 kg/m^3^, *Cp* ≈ 750 J/(kg · K), *k* ≈ 9 W/(m · K). Under high-energy beam processing conditions (P = 230 W, V = 1.25 m/s), the TiO_2_ particles experience an extremely high heating rate of approximately 10^6^ K/s. The calculations indicate that nanoscale particles (*r* = 10–100 nm) reach thermal equilibrium within approximately 10^−11^~10^−9^ s, far shorter than the characteristic melt pool residence time. The 20 nm-diameter tracers used in this study correspond to the lower end of this range. For a particle with a radius of 1 μm, the corresponding timescale is approximately 10^−7^ s, which remains shorter than the characteristic melt pool residence time of approximately (∼10^−5^ s) [32]. These estimates characterize the timescale of internal temperature redistribution within the particles. Their thermal exposure during transport is evaluated using the trajectory-dependent temperature histories below.

The TiO_2_ melting temperature of 2116 K was used as a reference for comparing thermal exposure along the selected trajectories. Figure 2c presents the temperature histories associated with tracers initially located at different depths beneath the laser center. Temperatures above 2116 K were recorded for most selected tracers, with peak values exceeding 3500 K near the melt pool center. The differences in peak temperature and subsequent cooling reflect the spatial variation in thermal exposure experienced by the tracers during transport [33].

To relate particle transport to the thermal histories, Figure 2d,e show the three-dimensional trajectories of representative particles initially located at different transverse positions and depths, respectively. The selected particles are highlighted in Figure 2b. At each recorded time, the local cell temperature was extracted at the instantaneous position of each tracer and used to color the corresponding point on its trajectory, thereby providing a spatially resolved representation of the thermal history experienced by each tracer during transport.

Figure 2d compares particles 51, 52, and 53, initially located at Z = 130 μm and Y = 90, 110, and 130 μm, respectively. Despite starting at the same initial depth, these particles follow distinctly different paths. Particle 51 undergoes a pronounced downward deflection, whereas particles 52 and 53 follow shorter recorded paths with smaller vertical excursions. The different paths and temperature variations along them indicate that the initial lateral position affects the thermal environments sampled during transport, even when the initial depth is unchanged.

Figure 2e compares particles 36, 44, and 52, initially located at Y = 110 μm and Z = 110, 120, and 130 μm, respectively. Particles 36 and 44 exhibit more strongly curved rearward trajectories and greater vertical displacement than particle 52. The corresponding temperature histories in Figure 2c show that all three particles experience temperatures above the TiO_2_ melting point, although their peak temperatures and subsequent cooling histories differ.

The combined analysis of Figure 2c–e shows that the thermal histories experienced by the tracers depend on both their initial spatial locations and their subsequent transport through the melt pool. Although rapid internal heat conduction promotes temperature homogenization within nanoscale TiO_2_ particles, the local thermal conditions encountered along their paths remain nonuniform. Assessment of the conditions for particle melting therefore requires the thermal history along each transport path, rather than the peak melt pool temperature alone.

**Figure 3:**
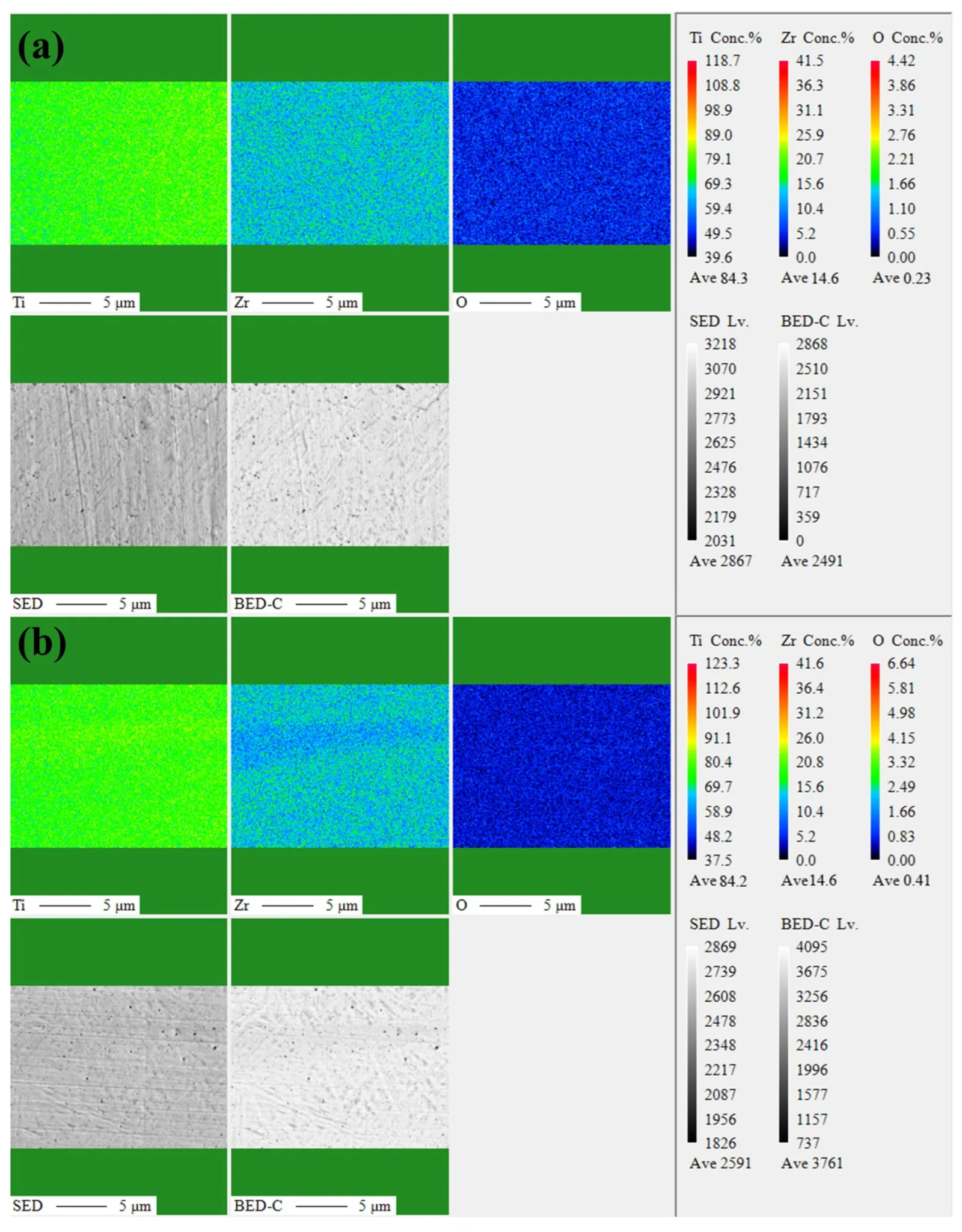
EPMA elemental maps and electron images of the as-built samples: (**a**) Ti-15Zr-0.2O; (**b**) Ti-15Zr-0.4O. Scale bars: 5 μm.

Figure 3 presents the EPMA elemental maps of the Ti-15Zr-0.2O and Ti-15Zr-0.4O samples. Oxygen signals are detected within the mapped regions of both samples. These maps describe local elemental distributions but do not establish complete TiO_2_ dissolution or the absence of residual oxides.

Overall, the tracer analysis shows that thermal exposure varies with both the initial particle position and subsequent transport through the melt pool. The trajectory-dependent temperature histories provide a spatially resolved description of this variation. This analysis provides a thermal and transport basis for considering the influence of dissolved oxygen on the surface tension temperature coefficient in the following section.

### 3.3. Regulation of the Surface Tension Temperature Coefficient by Oxygen Content

Building on the particle thermal-response analysis in Section 3.2, the influence of dissolved oxygen on surface tension was incorporated into the model. The particle histories indicate that much of the TiO_2_ population experiences temperatures sufficient for melting, providing the thermal basis for subsequent dissolution and oxygen release. For the following driving-force analysis, oxygen was treated through the prescribed surface tension relation, while the effects of residual TiO_2_ particles and dissolved oxygen on bulk properties, including density, viscosity, and thermal conductivity, were neglected. This treatment isolates the influence of oxygen-dependent surface tension on melt pool flow.

Since the melt pool flow is mainly governed by surface tension gradients, together with the recoil pressure induced by metal evaporation, this study focuses on the dynamic regulation of surface tension by oxygen. As a typical surface-active element, oxygen exhibits strong adsorption sensitivity at the liquid–gas interface and significantly modifies the temperature coefficient of surface tension. For a metallic ternary system containing surface-active solutes *i* and *j*, the surface tension can be described based on the Langmuir-Gibbs [34] adsorption isotherm as follows [35].

(19) $$\begin{array}{c} \sigma =\sigma^{o}+\sigma_{T}\left(T-T_{m}\right)-\Gamma_{i}\mathrm{RT}\ln\left(\frac{1+K_{i}a_{i}+K_{j}a_{j}}{1+K_{j}a_{j}}\right)-\Gamma_{j}\mathrm{RT}\ln\left(\frac{1+K_{i}a_{i}+K_{j}a_{j}}{1+K_{i}a_{i}}\right) \end{array}$$

where: *σ^o^* = 1.680 N m^−1^ is the surface tension of the pure metal at its melting point; *σ_T_* = −2.4 × 10^−4^ N m^−1^K^−1^ represents the temperature coefficient of surface tension; *T* is the melt temperature, and *T_m_* = 1950 K is the melting point of the material; *R* = 8.314 J·mol^−1^ · K^−1^ is the universal gas constant; *Γ_i_* is the saturated surface excess of solute *i* at the interface; *K_i_* is the equilibrium constant associated with the adsorption behavior, as defined in Equation (20); and *a_i_* is the thermodynamic activity of solute *i* in the melt.

(20) $$\begin{array}{c} K_{i}=k_{i}\exp\left(-\frac{\Delta H_{i}}{\mathrm{RT}}\right) \end{array}$$

where *k_i_* is a constant associated with the entropy of segregation, and Δ*H_i_* is the standard adsorption enthalpy of solute *i*. Based on the above model, the temperature dependence of the surface tension of Ti-15Zr-*x*O alloys with different oxygen contents can be determined.

Following the surface-segregation formulation of Chia et al. [20], the surface tension of the Ti-Zr-O melt was described using a pure-Ti reference and effective contributions from O and Zr. Experimental surface-tension data for Ti-Zr alloys [36] and thermodynamic assessments of the Ti-Zr-O system [37] were considered in selecting the model parameters. The saturation surface excesses were *Γ_O_* = 1.708 × 10^−5^ mol m^−2^ and *Γ_Zr_* = 1.5 × 10^−5^ mol m^−2^. The corresponding pre-exponential factors in Equation (20) were *k_O_* = 0.0138 and *k_Zr_* = 0.012, with standard adsorption enthalpies of Δ*H_O_* = −152 kJ mol^−1^ and Δ*H_Zr_* = −120 kJ mol^−1^. An ideal-solution approximation was adopted, with the activity coefficients set to unity. The activities of O and Zr were therefore approximated by their mole fractions, calculated from the alloy mass fractions as:

(21) $$\begin{array}{c} a_{i}=\frac{w_{i}/M_{i}}{w_{\mathrm{Ti}}/M_{\mathrm{Ti}}+w_{O}/M_{O}+w_{\mathrm{Zr}}/M_{\mathrm{Zr}}} \end{array}$$

where *w_i_* and *M_i_* denote the mass fraction and molar mass of component *i*, respectively. These parameters and the calculated activities were used to evaluate the temperature-dependent surface tension of Ti-15Zr-*x*O melts.

**Figure 4:**
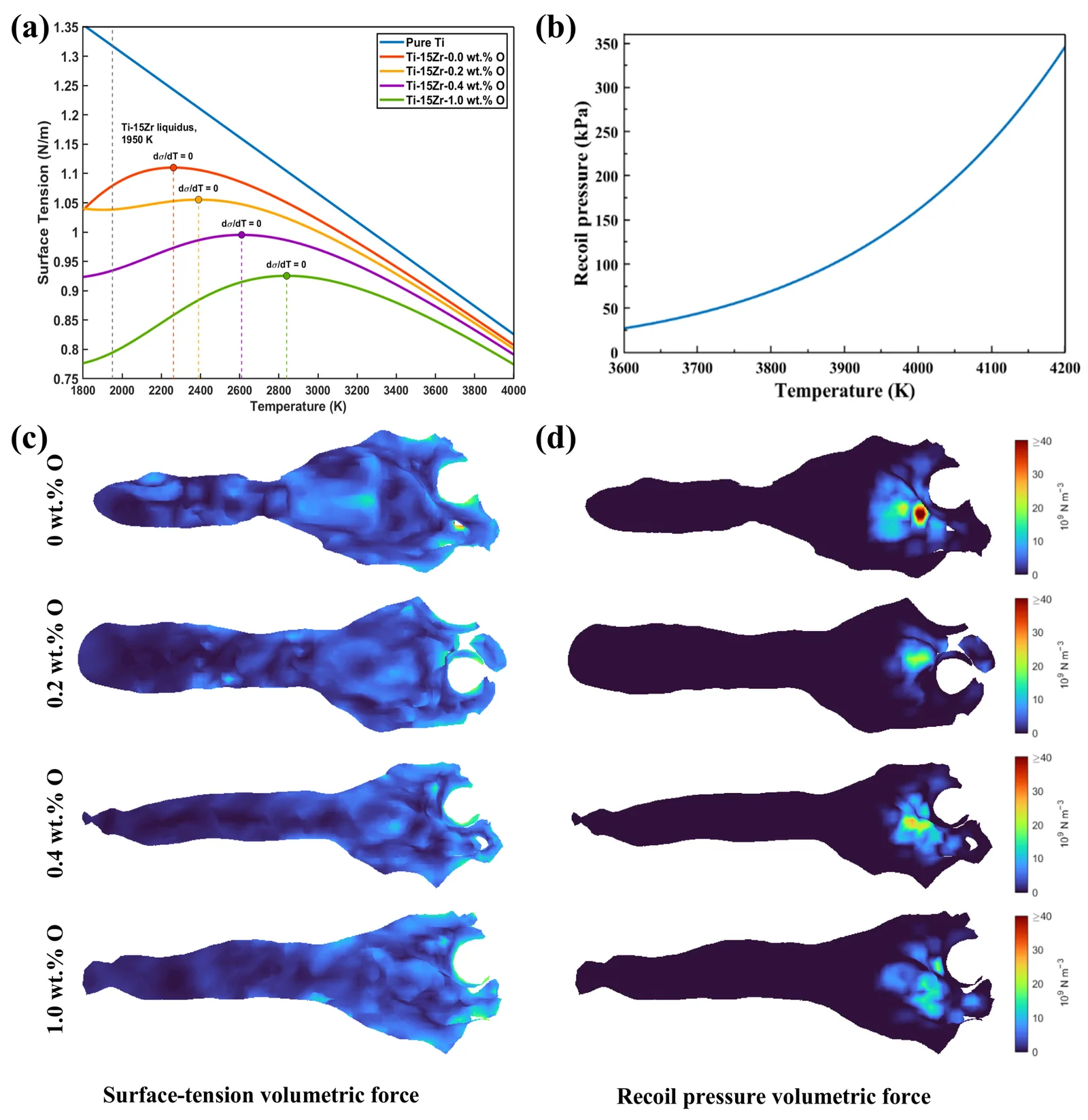
Temperature dependence and spatial distribution of the interfacial driving forces: (**a**) calculated surface tension of Ti-15Zr-*x*O alloys; (**b**) recoil pressure as a function of temperature in the high-temperature range shown; (**c**) surface-tension-induced volumetric force distribution; (**d**) recoil-pressure-induced volumetric force distribution.

To systematically investigate the regulatory effect of oxygen content on melt pool flow behavior and microstructural evolution, four representative oxygen concentrations were selected in this study: 0 wt.%, 0.2 wt.%, 0.4 wt.%, and 1.0 wt.%. The 0 wt.% condition was used as the baseline reference, while 0.2 wt.% and 0.4 wt.% corresponded to the experimental conditions. The 1.0 wt.% O condition was included solely as a theoretical upper-bound case to evaluate the limiting effects of oxygen enrichment on melt pool behavior and to elucidate the asymptotic evolution of oxygen-induced surface tension characteristics, rather than to represent a realistic LPBF processing condition. This parameter design spans low-, intermediate-, and high-oxygen regimes, enabling systematic investigation of the nonlinear effects of oxygen content on melt pool behavior. Figure 4a presents the calculated surface tension-temperature relations, with pure Ti included solely as a reference and not as a separate melt pool simulation case. Unlike the monotonic decrease for pure Ti, the Ti-15Zr-*x*O curves exhibit composition-dependent maxima that shift toward higher temperatures as the oxygen content increases.

For Ti-15Zr with 0 and 0.2 wt.% O, the surface tension maxima occur at approximately 2250 and 2400 K, respectively. Both curves therefore contain positive- and negative-slope intervals, rather than remaining negative over the entire temperature range. Above the respective maxima, the negative surface tension temperature coefficient favors transport from hotter toward cooler surface regions, consistent with the predominantly outward flow discussed below. The relatively flat portion of the 0.2 wt.% O curve near its maximum weakens the local thermocapillary driving force and indicates a transitional response.

**Figure 5:**
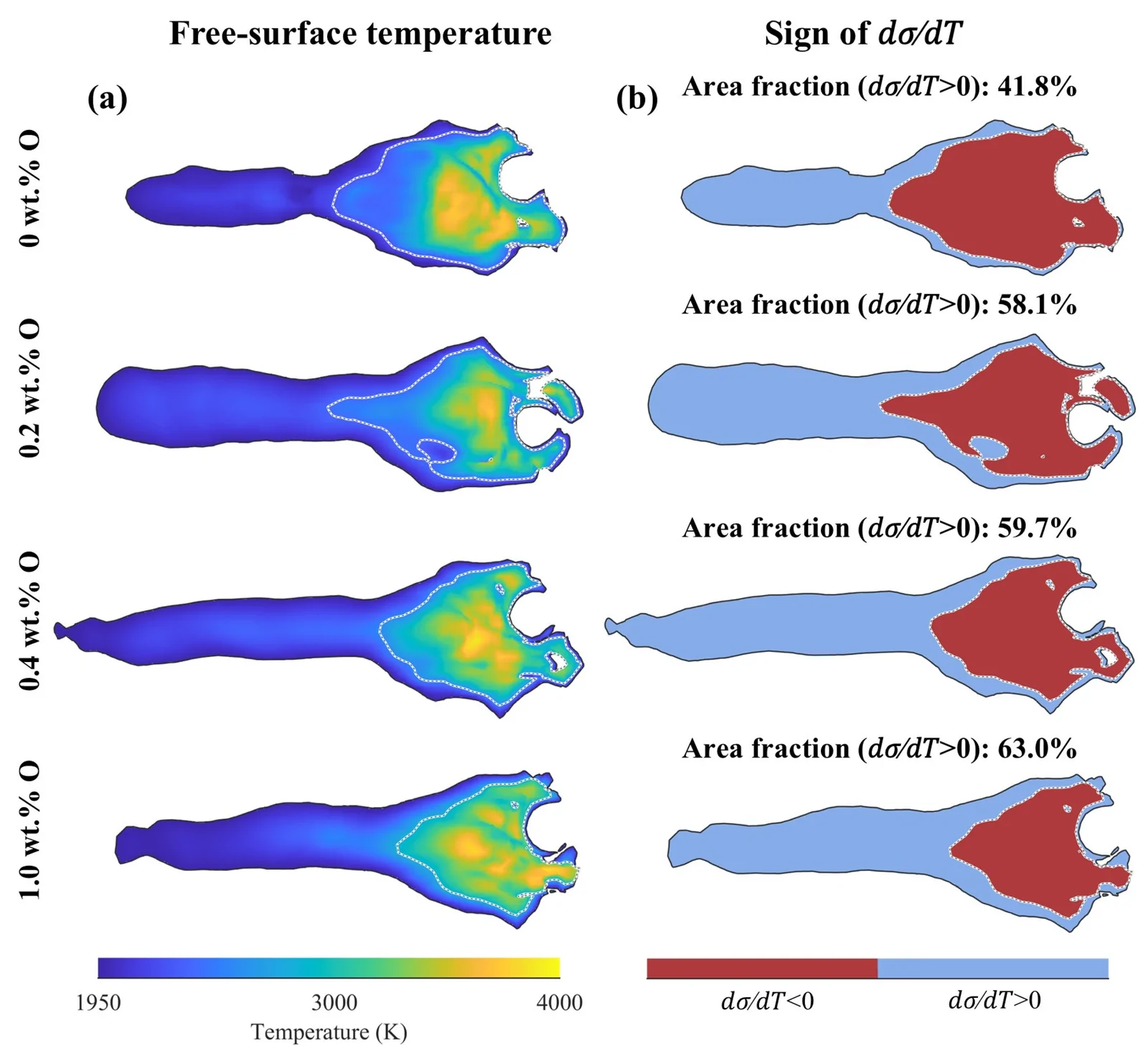
Top views of the melt-pool free surface at different oxygen contents: (**a**) temperature distribution; (**b**) schematic map of *dσ*/*dT*.

When the oxygen content is further increased to 0.4 wt.% and 1.0 wt.%, a distinct reversal of the surface tension slope is observed. Within the positive-slope interval below the composition-dependent turning temperature T*, the surface tension increases with temperature (*dσ*/*dT* > 0), favoring inward flow along the free surface. The hottest central region retains *dσ*/*dT* < 0 (Figure 5). Comparison between the 0.4 wt.% and 1.0 wt.% curves further reveals that the magnitude of the positive surface tension gradient increases with higher oxygen concentration, thereby enhancing the centripetal constraint on melt convection.

The return to a negative slope at higher temperatures reflects the temperature-dependent balance between solute adsorption and the intrinsic decrease in surface tension of the solvent. As adsorption becomes less favorable with increasing temperature, the intrinsic negative temperature dependence can again dominate [18]. Thus, the maxima in Figure 4a are composition-dependent transition temperatures.

Figure 4b shows the temperature dependence of recoil pressure within the evaporation-dominated temperature range. The recoil pressure increases increasingly rapidly with temperature, indicating that localized overheating can generate a highly concentrated evaporation-induced load. Within the present model, oxygen does not explicitly enter the recoil-pressure formulation, and the thermophysical parameters governing the pressure-temperature relation are assumed to remain unchanged over the investigated oxygen range. Therefore, the same recoil-pressure-temperature relation is applied to all oxygen contents. It should be noted, however, that the actual spatial distribution of recoil pressure may still vary indirectly through oxygen-induced changes in the local temperature field.

The spatial distributions in Figure 4c,d distinguish the regions over which the two mechanisms act. The surface-tension-induced volumetric force extends over a broad portion of the free surface, whereas appreciable recoil-pressure forcing is concentrated around the laser interaction region. The plotted quantities are momentum-source magnitudes per unit volume, rather than surface pressure or flow direction. Their different spatial extents motivate the decoupled comparison below, which examines how localized recoil forcing interacts with oxygen-dependent surface transport.

Figure 5 shows the free-surface temperature distributions and the corresponding spatial distributions of the sign of *dσ*/*dT*. Regions with positive and negative temperature coefficients coexist at all four oxygen contents. The hotter central region generally exhibits (*dσ*/*dT* < 0), whereas positive values occur over cooler peripheral and trailing regions. As the oxygen content increases from 0 to 0.2, 0.4, and 1.0 wt.%, the projected-area fraction with (*dσ*/*dT* > 0) increases from 41.8% to 58.1%, 59.7%, and 63.0%, respectively. Together with the surface-tension–temperature curves in Figure 4a, these results show that oxygen content changes the spatial extent of regions with opposite thermocapillary responses. This spatial redistribution affects the thermal contribution to the local Marangoni stress and provides a basis for interpreting the flow patterns discussed below. The positive-slope area fraction changes by only 1.6 percentage points between 0.2 and 0.4 wt.% O. The oxygen-dependent flow changes are therefore interpreted using these fields together with the coupled velocity distributions.

### 3.4. Oxygen-Dependent Melt Pool Flow Under Solid Substrate Conditions

Building on the oxygen-dependent surface-tension relation described in Section 3.3, this section examines how surface tension and recoil pressure interact to modify melt-pool flow on an idealized solid substrate. Three configurations were compared: surface tension with recoil pressure omitted, recoil pressure with surface tension omitted, and the coupled model. The surface-tension contribution includes both normal capillary forces and tangential Marangoni stresses. These comparisons examine the roles of the two interfacial mechanisms in melt-pool circulation and free-surface evolution. The simulations used identical laser power and scanning speed, with prescribed oxygen contents of 0, 0.2, 0.4, and 1.0 wt.%.

During simulation, the central cross-section at the quasi-steady stage of the melt pool was selected for quantitative analysis. Flow-field and geometric data were extracted beginning at x = 400 μm along the scanning direction to quantify the effect of oxygen content on the evolution of melt pool morphology and flow-field structure.

**Figure 6:**
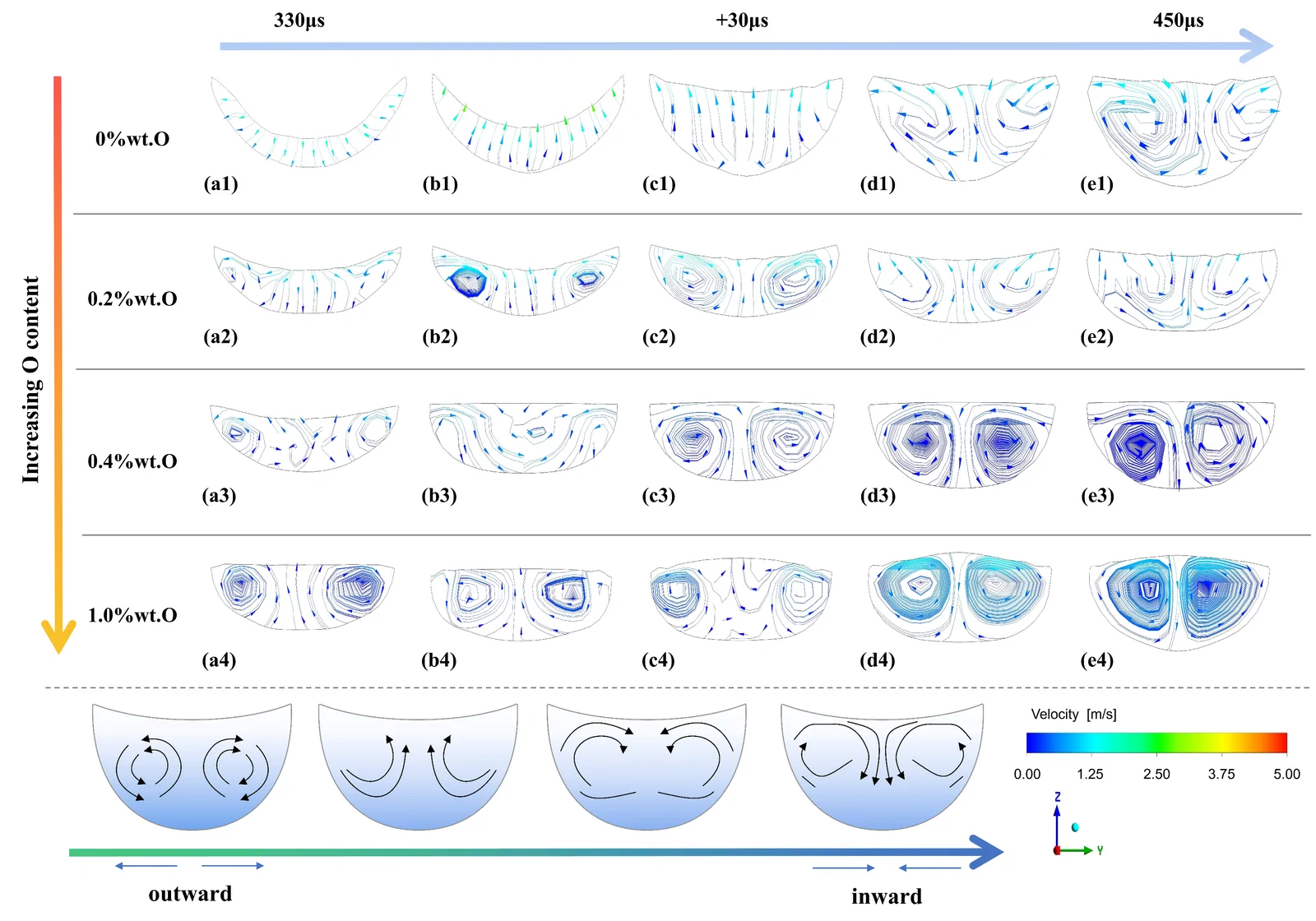
Evolution of melt pool flow and morphology driven solely by surface tension: (**a1**–**e1**) 0 wt.% O; (**a2**–**e2**) 0.2 wt.% O; (**a3**–**e3**) 0.4 wt.% O; (**a4**–**e4**) 1.0 wt.% O.

Figure 6 illustrates the evolution characteristics of the internal melt pool flow field under the single driving force of surface tension alone. Under this condition, the temperature-dependent surface tension gradient along the free surface governs the direction of the Marangoni shear stress. Combined with the surface tension evolution shown in Figure 4, it can be seen that in the low-oxygen range (0–0.2 wt.% O), the corresponding regime is dominated by a negative surface tension gradient (*dσ*/*dT* < 0). Under this condition, the surface tension decreases progressively from the low-temperature peripheral region toward the high-temperature laser interaction zone, thereby driving the molten metal to flow outward from the center toward the edges along the free surface. This produces the outward divergent flow pattern shown in Figure 6. Macroscopically, this promotes lateral spreading of the melt pool, producing a relatively wide and shallow morphology.

In contrast, under high-oxygen conditions (0.4–1.0 wt.% O), the surface tension increases with temperature (*dσ*/*dT* > 0) within the positive-slope interval below T*. In these surface regions, the thermocapillary stress drives molten metal toward hotter regions, promoting inward surface transport [38]. As shown in Figure 6, the inward flow is accompanied by downward circulation and a closed double-vortex structure. This flow pattern limits lateral spreading and promotes a narrower, deeper melt pool. The observed transition from outward spreading to inward circulation is consistent with oxygen-dependent changes in the surface tension temperature coefficient [20].

**Figure 7:**
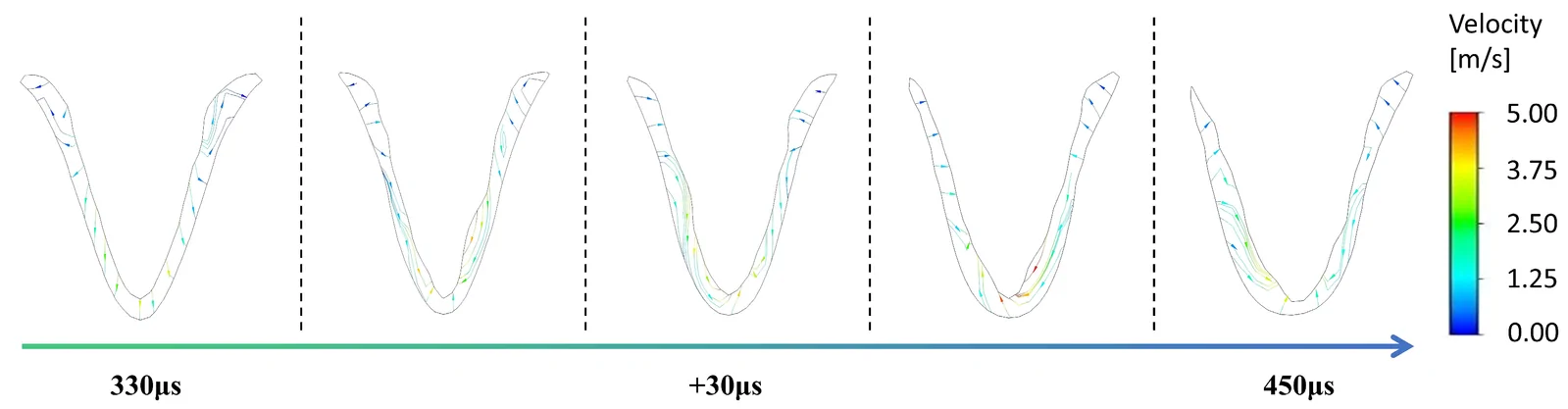
Evolution of melt pool flow and morphology driven solely by recoil pressure.

To isolate evaporation-induced forcing, surface tension was omitted in the recoil-pressure-only calculation. Figure 7 presents the cross-sectional flow and surface morphology from 330 to 450 μs. During laser interaction, recoil pressure acts approximately normal to the local free surface and displaces molten metal from the evaporating region. This localized loading produces a pronounced central depression and promotes melt redistribution into the deeper region, consistent with the role of recoil pressure in deep-penetration melting [39].

Unlike the surface-tension-driven flow field, the recoil-pressure-driven flow shows high consistency across all oxygen concentrations. The flow topology changes only slightly with oxygen content. Physically, the magnitude of recoil pressure mainly depends on the local surface temperature induced by laser energy input, together with the intrinsic thermophysical properties of the Ti-15Zr alloy. Since trace oxygen addition (0–1.0 wt.% O) as a solute within the investigated range produces only negligible changes in the thermophysical properties governing evaporation, the recoil pressure remains primarily controlled by the laser-induced surface temperature. Consequently, the overall recoil pressure distribution changes only slightly.

**Figure 8:**
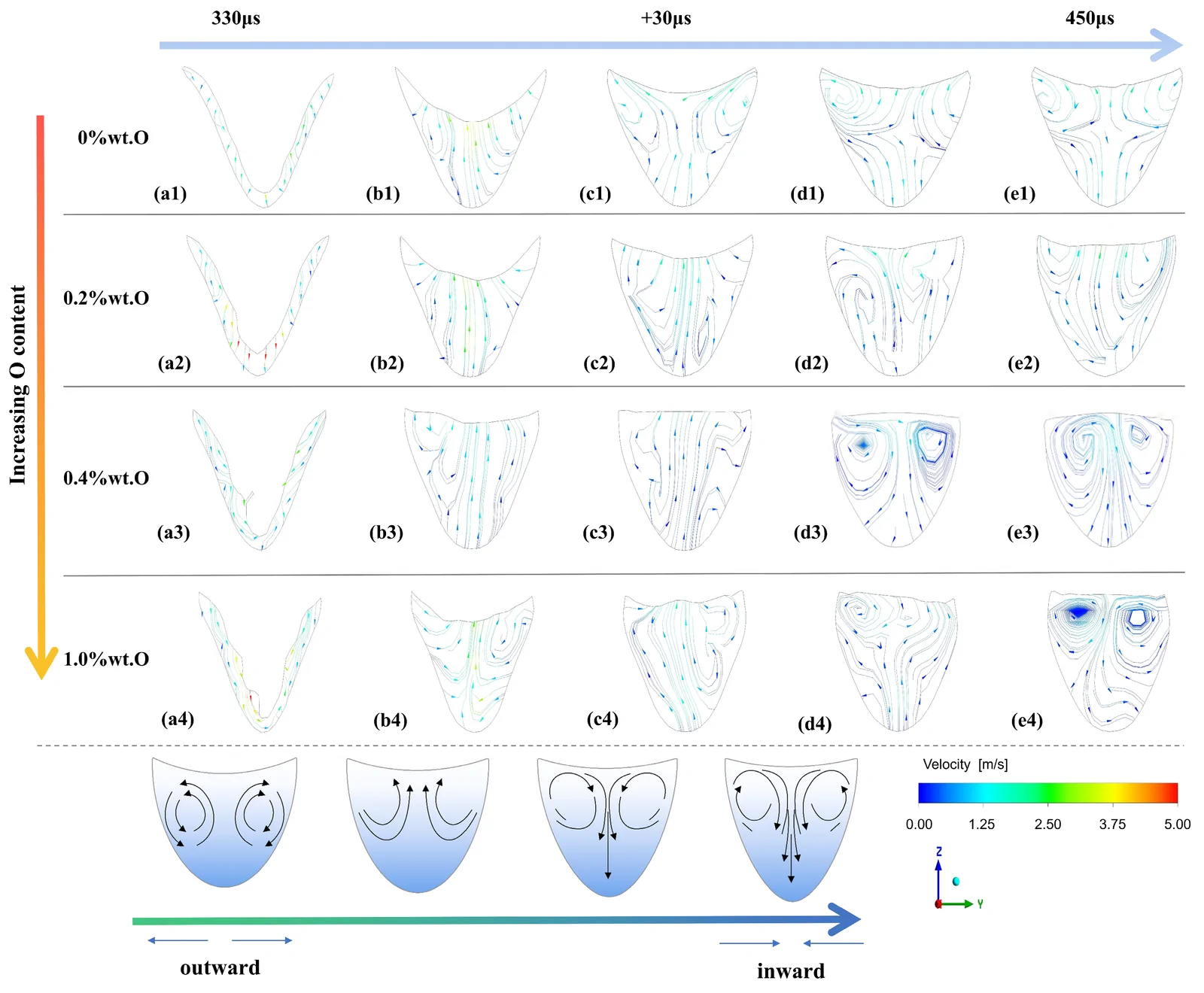
Evolution of melt pool flow and morphology under the coupled effects of surface tension and recoil pressure: (**a1**–**e1**) 0 wt.% O; (**a2**–**e2**) 0.2 wt.% O; (**a3**–**e3**) 0.4 wt.% O; (**a4**–**e4**) 1.0 wt.% O.

The persistence of the V-shaped depression in Figure 7 results from the imposed decoupling. Once the laser moves beyond the section, local cooling reduces evaporation and the associated recoil pressure; the remaining depression does not imply an unchanged recoil load. Both the curvature-induced capillary force and tangential Marangoni stress are absent from this calculation. In particular, the capillary restoring mechanism that normally promotes depression closure as recoil pressure decreases is suppressed [11]. Consequently, the free surface does not return to its initial, nearly flat configuration within the time interval shown. Inertia, gravity, and solidification can still influence the residual shape. Figure 7 provides an idealized force-removal comparison; the role of recoil pressure is assessed using the coupled results in Figure 8.

Restoring surface tension in the coupled model allows both capillary relaxation and oxygen-dependent Marangoni transport to interact with recoil-pressure-driven flow. Figure 8 compares the resulting melt pool evolution at oxygen contents of 0–1.0 wt.%. Recoil pressure continues to promote central penetration, while the surface tension gradient changes the redistribution of molten metal and the internal circulation [40]. The comparison therefore distinguishes the role of recoil pressure in forming the central depression from the role of surface tension in redistributing liquid and modifying the depression during its subsequent evolution.

However, the oxygen-induced change in surface tension gradient still significantly modifies the local internal flow structure. As shown in Figure 8, increasing oxygen content fundamentally changes the relative flow directions induced by surface tension and recoil pressure, causing their interaction to evolve from competition to cooperative reinforcement. Under low-oxygen conditions, the negative surface tension gradient drives molten metal outward from the center toward the edges. This outward Marangoni shear competes with the downward recoil-pressure jet in the upper region of the melt pool. Because the outward Marangoni flow continuously transports molten metal away from the laser center along the free surface, it reduces the amount of molten metal available for downward transport beneath the laser spot, thereby weakening the downward circulation established by recoil pressure. Geometrically, this competition manifests as divergent streamlines, an open flow field, and a relatively wide melt pool opening [41].

By contrast, in the high-oxygen range, the positive surface tension gradient drives molten metal inward from the periphery toward the center. This inward Marangoni flow acts synergistically with the downward recoil-pressure jet in the upper region of the melt pool. Because the inward Marangoni flow continuously transports molten metal toward the laser center along the free surface, it supplies additional molten metal for downward transport beneath the laser spot, thereby strengthening the downward circulation established by recoil pressure. Geometrically, this cooperation manifests as a pronounced closed double-vortex flow inside the melt pool. This cooperative flow not only enhances heat transport toward the deeper region of the melt pool but also significantly reduces the opening width at the melt surface, ultimately forming a deeper and narrower melt pool in the transition mode [16].

The transition in Marangoni flow topology occurs within an oxygen concentration range of approximately 0.2–0.4 wt.%, where the flow topology changes from outward divergence to inward convergence. These results demonstrate that oxygen fundamentally regulates melt pool flow by modifying the balance between Marangoni convection and recoil pressure under idealized solid-substrate conditions. The following section extends the above analysis from the idealized solid-substrate model to realistic powder-bed conditions, thereby evaluating the robustness of the identified driving-force interaction mechanism.

### 3.5. Oxygen-Dependent Melt Pool Flow Behavior in a Discrete Powder-Bed Condition

After clarifying the oxygen-dependent flow mechanisms under the idealized solid-substrate condition, the analysis was extended to a discrete powder bed. Previous simulations of electron beam powder bed fusion have linked the coalescence of randomly distributed powder particles to track irregularity and defect development [42]. To account for these powder-scale effects, a high-fidelity discrete powder-bed model was employed to examine whether the identified interaction between Marangoni convection and recoil pressure remains evident during single-track formation when powder melting and free-surface evolution are explicitly resolved.

**Figure 9:**
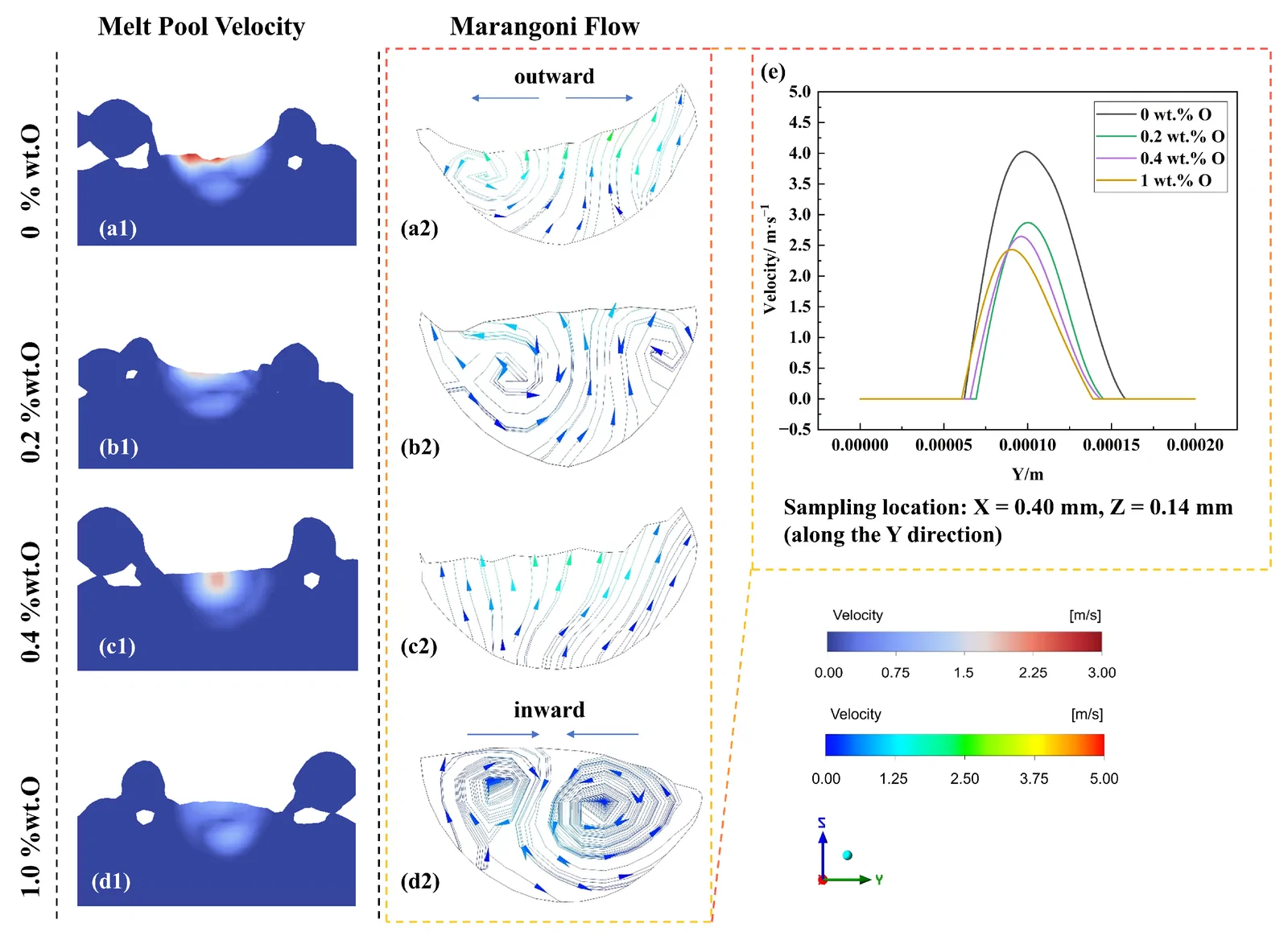
Quasi-steady melt pool flow characteristics under discrete powder-bed conditions (Y-Z section): (**a1**–**d1**) velocity field; (**a2**–**d2**) flow streamlines; (**e**) transverse surface velocity distributions at different oxygen contents.

Figure 9 presents the thermofluid characteristics in the melt pool cross-section (Y-Z plane) at the quasi-steady state (t = 0.00033 s), including velocity distributions, Marangoni streamlines, and transverse surface velocity profiles. Despite the additional complexities introduced by powder melting and free-surface evolution, the overall flow topology remains highly consistent with that predicted under the idealized solid-substrate condition. The oxygen-dependent transition from outward-diverging flow to inward-converging circulation is preserved, indicating that the governing flow mechanism identified in Section 3.4 remains applicable under realistic LPBF conditions [18].

In the low-oxygen range, Figure 9(a2,b2) show outward surface transport together with local recirculation. The negative surface tension temperature coefficient over the relevant surface regions favors transport from the hotter interaction zone toward the cooler periphery. This lateral redistribution promotes a relatively wide upper opening and a broad U-V-shaped melt pool, consistent with the solid-substrate comparison.

In contrast, increasing oxygen content causes the melt pool to transition to an inward-converging flow mode, consistent with the mechanism established in Section 3.4. As shown in Figure 9(c2,d2), the positive surface tension temperature gradient drives molten metal from the pool periphery toward the laser center, where it cooperates with the recoil-pressure-driven downward flow to establish a stable closed double-vortex circulation. This inward flow pattern effectively confines the molten metal beneath the laser spot, resulting in a deeper and narrower melt pool.

To quantitatively characterize the oxygen-dependent flow evolution, Figure 9e compares the transverse surface velocity profiles under quasi-steady conditions. The spatial span of the velocity curve represents the effective flow width, while the peak velocity corresponds to the intensity of surface convective heat transfer [43]. As oxygen content increases, both the lateral extent of surface flow and the peak surface velocity decrease continuously. Under oxygen-free conditions, the velocity profile spans the widest region, indicating pronounced outward flow and extensive lateral spreading. With increasing oxygen content, the velocity profile progressively contracts toward the laser center, reflecting the enhanced confinement imposed by inward Marangoni convection. Consequently, the melt pool surface width decreases systematically with increasing oxygen content [44].

The peak surface velocity also decreases markedly with increasing oxygen content, from approximately 4.0 m/s under oxygen-free conditions to about 2.4 m/s at 1.0 wt.% O. This reduction indicates that inward Marangoni convection progressively weakens the net surface momentum by partially offsetting the recoil-pressure-driven flow, thereby suppressing surface convection intensity.

**Figure 10:**
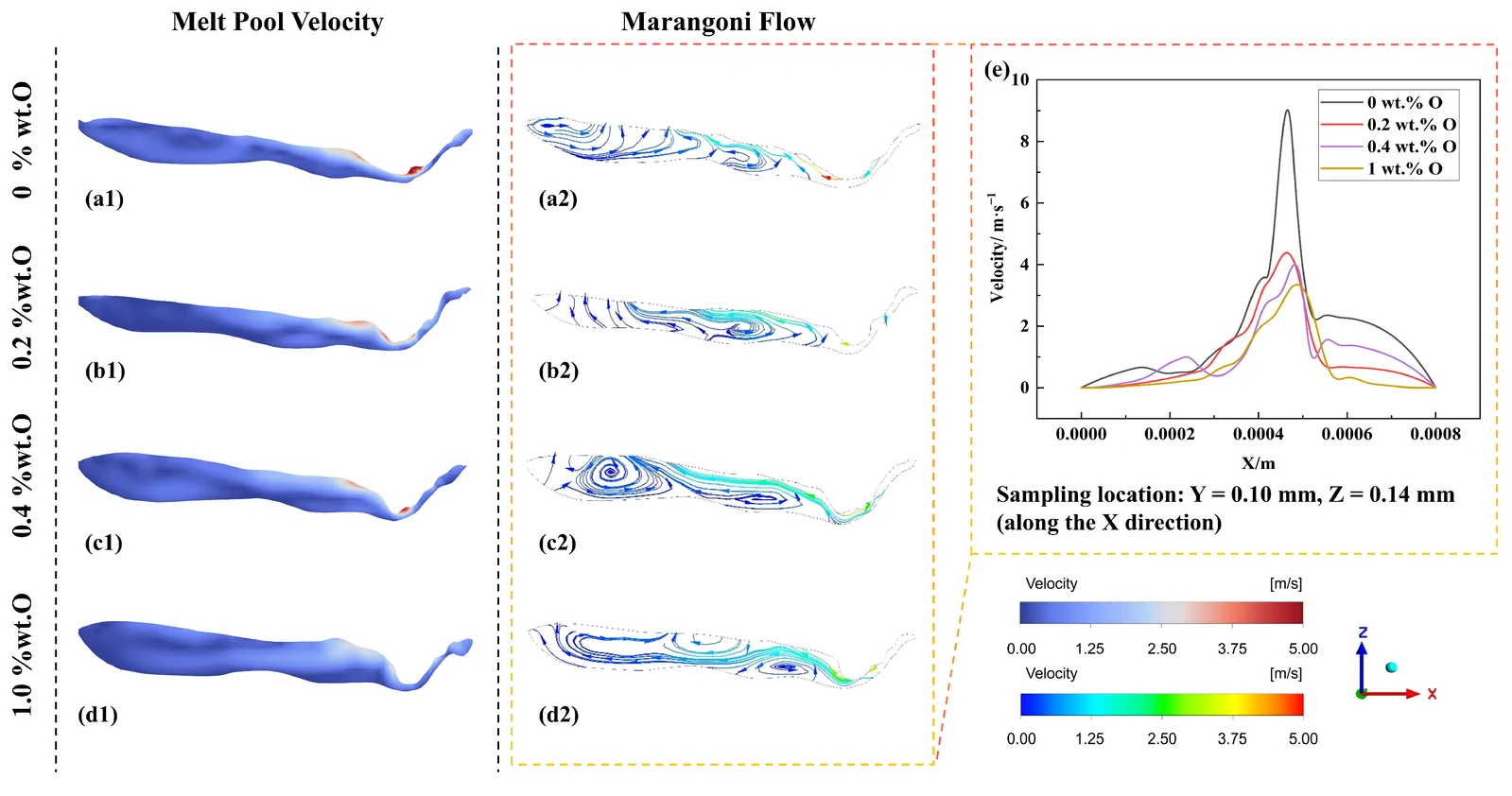
Quasi-steady melt pool flow characteristics under discrete powder-bed conditions (X-Z section): (**a1**–**d1**) velocity field; (**a2**–**d2**) flow streamlines; (**e**) longitudinal surface velocity distributions at different oxygen contents.

Besides modifying the transverse flow behavior, oxygen content also markedly alters thermofluid transport in the longitudinal X-Z plane. Figure 10 presents the longitudinal flow field and the corresponding surface velocity profiles along the melt pool centerline under different oxygen contents. Similar to the transverse section, two distinct longitudinal flow modes are observed with increasing oxygen content.

In the low-oxygen range, as shown in Figure 10(a2,b2), the surface streamlines remain smooth and continuously extend backward. Driven by the negative surface tension gradient, the surface fluid is continuously transported from the high-temperature region at the front of the vapor depression toward the low-temperature rear region. This backward-directed convection significantly enhances heat transport along the scanning direction, causing an obvious elongation of the melt pool. Moreover, because the surface flow direction is consistent with the fluid displacement induced by recoil pressure, the rear flow field remains relatively stable without significant vortex disturbance.

Under high-oxygen conditions, the longitudinal flow undergoes significant topological reconstruction. As shown in Figure 10(c2,d2), the reversal of the surface tension temperature gradient redirects the surface flow toward the laser center, fundamentally altering the longitudinal momentum transport established under low-oxygen conditions. Transport toward the laser interaction region and return flow behind the vapor depression redistribute molten metal within the middle and trailing regions of the pool. The positions and extent of the recirculating structures change accordingly. This reverse flow subsequently interacts with the recoil-pressure-driven downward and backward flow, giving rise to a stable closed vortex in the middle-to-rear region of the melt pool. As a consequence, the continuous downstream transport of heat and molten metal is interrupted, leading to local accumulation beneath the rear wall of the vapor depression and thereby modifying the longitudinal temperature distribution and subsequent solidification behavior.

These flow characteristics are further confirmed by the surface velocity profiles in Figure 10e. Under oxygen-free conditions, the peak surface velocity near the laser center reaches approximately 9.0 m/s, mainly due to the synergistic acceleration produced by the outward Marangoni force and recoil pressure acting in the same direction. In contrast, under high-oxygen conditions, because inward Marangoni convection partially offsets the recoil-pressure-driven downstream transport, a pronounced mechanical counteraction occurs, significantly suppressing longitudinal momentum transport and reducing the peak velocity to approximately 3.0–4.0 m/s.

In addition, the nonlinear fluctuations appearing at the tail of the velocity curve under high-oxygen conditions correspond closely to the reverse recirculation vortex in the flow field, indicating that this recirculation structure intensifies local flow oscillations and increases flow instability in the tail region [45]. This observation further confirms that oxygen regulates not only the mean flow direction but also the stability of melt-pool convection.

To quantify the thermocapillary driving associated with the flow patterns described above, the Marangoni numbers were evaluated separately for the positive- and negative-slope branches of the surface-tension curves. They were defined as:

(22) $$\begin{array}{c} M_{1}=\frac{\sigma (T^{*})-\sigma (T_{l})}{T^{*}-T_{l}} \end{array}$$

(23) $$\begin{array}{c} M_{2}=\frac{\sigma (T_{\max})-\sigma (T^{*})}{T_{\max}-T^{*}} \end{array}$$

(24) $$\begin{array}{c} {\mathrm{Ma}}_{1}=\frac{M_{1}(T^{*}-T_{l})L}{\mu \;\alpha_{\mathrm{th}}} \end{array}$$

(25) $$\begin{array}{c} {\mathrm{Ma}}_{2}=\frac{M_{2}(T_{\max}-T^{*})L}{\mu \;\alpha_{\mathrm{th}}} \end{array}$$

Here, *T_l_* is the liquidus temperature, *T_max_* is the maximum melt-pool surface temperature, and T* is the temperature separating the positive- and negative-slope branches of the surface-tension curve. The reference length *L* was taken as half the mean solidified-track width within the stable scanning region. For this characteristic-number calculation, we use *μ* = 0.004 Pa s and *α*_th_ = 7.4074 × 10^−6^ m^2^ s^−1^. The calculated values are summarized in Table 4. These Marangoni numbers are characteristic estimates based on interval-averaged surface-tension slopes. They do not constitute a force balance or quantitatively separate Marangoni and recoil-pressure contributions to the coupled flow.

As shown in Table 4, |*Ma*_2_| decreases from 252.7 to 112.9 as the oxygen content increases from 0 to 1.0 wt.%, indicating a reduction in the characteristic driving of the negative-slope branch. *Ma*_1_ initially decreases from 32.2 to 16.5 and then increases to 55.1 and 113.4 at 0.4 and 1.0 wt.% O, respectively. At the highest oxygen content, the positive-slope branch reaches a magnitude comparable to that of the negative-slope branch. Combined with the surface-temperature distributions, this change indicates a growing role of inward thermocapillary transport at higher oxygen contents. This trend agrees with the reduced transverse flow extent in Figure 9 and the reverse circulation behind the laser interaction region in Figure 10. These results are consistent with the observed flow changes discussed above, in which oxygen alters surface transport and its interaction with recoil-driven circulation, suppressing lateral melt spreading and producing narrower tracks.

The coupled powder-bed simulations retain the main oxygen-dependent flow features observed under the idealized solid-substrate condition. Oxygen-dependent surface tension modifies transverse melt spreading and longitudinal recirculation while interacting with recoil-driven transport. These changes are associated with the predicted track-width and depth variations analyzed in the following section.

### 3.6. Effect of Oxygen Content on Melt Pool Geometry

The oxygen-dependent melt pool flow behavior established in the previous section ultimately governs the geometry of the solidified melt track. Therefore, the morphology, width, and depth of single tracks under different oxygen contents were quantitatively analyzed. It should be emphasized that the velocity fields and streamlines discussed in the previous sections represent transient melt pool dynamics during laser irradiation, whereas the present analysis focuses on the final geometry of the solidified melt track after solidification.

During single-track scanning, the melt pool is not fully established at the beginning of the scan and is affected by the termination of heat input near the end, resulting in noticeable fluctuations in track width. To minimize the influence of these transient regions, in addition to the average width of the entire melt track, the average width within the stable scanning region (0.20–0.40 mm) was also evaluated to characterize the representative melt track width under quasi-steady melting conditions.

**Figure 11:**
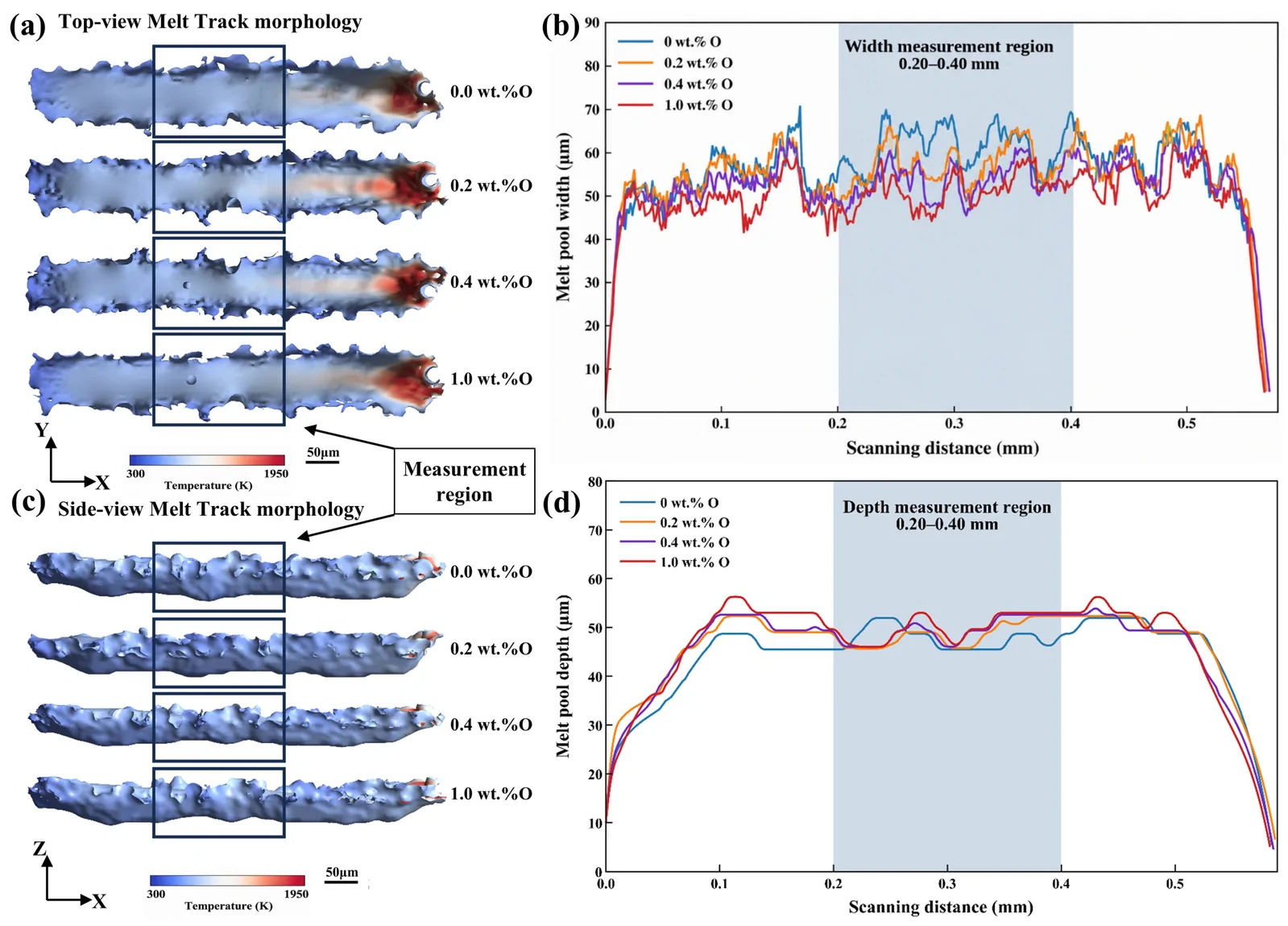
Three-dimensional morphology of the single-track melt tracks at different oxygen contents under discrete powder-bed conditions: (**a**) top-view morphology; (**b**) melt track width along the scanning direction; (**c**) side-view morphology; (**d**) melt track depth along the scanning direction.

Figure 11a presents the three-dimensional morphology of the solidified single tracks together with the measurement region used for width evaluation. The rectangular area indicates the stable scanning region (0.20–0.40 mm) adopted for statistical analysis. As shown, slight edge fluctuations are observed under all oxygen conditions, which are mainly attributed to the discrete powder-bed morphology, local powder packing variations, and melt spreading behavior. Compared with the scan initiation and termination regions, the central region exhibits a more continuous morphology and relatively stable track width, making it more suitable for quantitative comparison.

Figure 11b further shows the variation in melt track width along the scanning direction. The track width increases rapidly during scan initiation and decreases near the scan termination, whereas only minor fluctuations are observed within the selected stable region. As the oxygen content increases from 0 wt.% O to 1.0 wt.% O, the mean track width decreases, indicating a progressive reduction in the lateral dimension of the solidified melt track.

Table 5 summarizes the mean track dimensions obtained over the whole track and the stable scanning region (0.20–0.40 mm). As the oxygen content increases from 0 to 1.0 wt.%, the whole-track mean width decreases from 56.11 to 49.59 μm, corresponding to a reduction of 11.6%. Within the stable scanning region, the mean width decreases from 61.32 to 51.23 μm, corresponding to a reduction of 16.5%. The narrowing trend is consistent across both evaluation regions.

Figure 11c,d show the corresponding side-view morphology and depth profiles. From 0 to 1.0 wt.% O, the whole-track mean depth increases from 43.71 to 46.89 μm, while the stable-region mean depth increases from 47.71 to 49.95 μm, corresponding to increases of 7.3% and 4.7%, respectively. The depth-to-width ratios calculated from the stable-region mean dimensions are 0.778, 0.847, 0.920, and 0.975 at 0, 0.2, 0.4, and 1.0 wt.% O, respectively. The geometric change is therefore characterized primarily by lateral narrowing, accompanied by a modest increase in depth. During laser irradiation, the vapor-depression depth increases from 12.33 to 17.04 μm, and the maximum surface temperatures range from approximately 3739 to 3896 K (Table 5). These temperatures exceed the boiling temperature of 3560 K listed in Table 1. In the model, this boiling temperature serves as the reference in the recoil-pressure and evaporative heat-loss relations, Equations (10) and (12). The surface temperature is determined by the local balance of laser heating, heat transport, and evaporative cooling in Equation (7), and is not capped at the boiling temperature.

Combined with the melt pool flow analysis presented in the previous sections, this geometric evolution can be directly associated with the oxygen-induced transition of Marangoni convection. Under low-oxygen conditions, outward surface flow promotes the lateral spreading of molten metal, resulting in wider melt tracks. As the oxygen content increases, the surface flow gradually reverses toward the melt pool center, suppressing lateral mass transport and consequently limiting the lateral expansion of the melt pool. Accordingly, the reduction in melt track width is consistent with the weakened lateral transport capability and can therefore be regarded as a geometric response consistent with the oxygen-induced transition in melt pool flow behavior. The role of Marangoni convection in shaping solidified tracks has also been demonstrated by numerical simulations of electron beam powder bed fusion [46]. In the present study, oxygen-induced changes in the surface tension gradient modify the interaction between Marangoni convection and recoil-driven flow, suppressing lateral molten-metal transport and producing narrower tracks under fixed laser power and scanning speed.

Overall, oxygen-dependent changes in melt flow reduce lateral spreading and produce narrower melt tracks, accompanied by a comparatively modest increase in depth under the investigated processing conditions.

## 4. Conclusions

This study investigated the role of oxygen in regulating surface tension, melt pool dynamics, and melt track geometry in LPBF Ti-15Zr alloys through numerical simulations supported by single-track validation experiments. The main conclusions are as follows.

(1) The estimated internal thermal-diffusion timescale of nanoscale TiO_2_ particles was approximately 10^−11^–10^−9^ s, much shorter than the characteristic melt pool residence time (~10^−5^ s). Most selected tracers encountered local temperatures above 2116 K, with peaks exceeding 3500 K near the melt pool center. Their peak temperatures and cooling histories depended on initial position and subsequent trajectory, linking particle thermal exposure to three-dimensional melt circulation.
(2) Increasing oxygen content modified the spatial distribution of the surface-tension temperature coefficient and reorganized the interaction between Marangoni convection and recoil-driven transport. Positive and negative coefficients coexisted on the free surface. As oxygen content increased from 0 to 1.0 wt.%, the top-view projected-area fraction with a positive coefficient increased from 41.8% to 63.0%, corresponding to an increase of 21.2 percentage points. In transverse sections, outward Marangoni flow at low oxygen contents competed with recoil-driven downward transport and promoted lateral spreading, whereas inward flow at higher oxygen contents reinforced downward circulation. In longitudinal sections, the reversed surface flow opposed recoil-driven rearward transport and promoted recirculation behind the vapor depression.
(3) These oxygen-dependent flow patterns remained evident under discrete powder-bed conditions and were accompanied by systematic changes in track geometry. At 230 W and 1.25 m/s, increasing oxygen content from 0 to 1.0 wt.% reduced the mean track width within the 0.20–0.40 mm region along the melt pool length from 61.32 to 51.23 μm (16.5%), while the mean depth increased from 47.71 to 49.95 μm (4.7%). The ratio of mean depth to mean width increased from 0.778 to 0.975. The geometric response to oxygen addition was therefore characterized primarily by lateral narrowing, accompanied by a comparatively modest increase in depth. The oxygen-dependent results at 230 W and 1.25 m/s are model predictions. Validation was limited to track width at 1.0 m/s, with a systematic overprediction of 5.6–7.5%, which is appreciable compared with the predicted narrowing.

## Figures and Tables

**Table 1:** Thermophysical properties and model parameters used in the simulations [27,28,29,30].

Symbol	Parameter	Unit	Value
*ρ_s_*	Solid density	kg m^−3^	4680
*ρ* *_l_*	Liquid density	kg m^−3^	4500
*c_s_*	Specific heat, solid	J K^−1^·kg^−1^	540
*c_l_*	Specific heat, liquid	J K^−1^·kg^−1^	750
*k_s_*	Thermal conductivity, solid	W m^−1^·K^−1^	20
*k_l_*	Thermal conductivity. liquid	W m^−1^·K^−1^	25
*μ*	Dynamic viscosity	Pa s	4 × 10^−3^
*T_s_*	Solidus temperature	K	1880
*T_l_*	Liquidus temperature	K	1950
*T_v_*	Boiling temperature	K	3560
*L_m_*	Latent heat of melting	J kg^−1^	2.9 × 10^5^
*L_v_*	Latent heat of vaporization	J kg^−1^	9.5 × 10^6^
*η*	Laser absorptivity	-	0.6
*A_mush_*	Mushy-zone resistance coefficient	kg m^−3^ s^−1^	1.0 × 10^7^
*c_k_*	Mushy-zone regularization constant	-	0.001
*c_n_*	DEM normal damping coefficient	kg s^−1^	8.257 × 10^−6^
*c_t_*	DEM tangential damping coefficient	kg s^−1^	3.783× 10^−6^
*μ* *_f_*	DEM dynamic friction coefficient	-	0.28
*k_n_*	DEM normal stiffness	N m^−1^	8.49 × 10^−2^

**Table 2:** Mesh sensitivity analysis.

O Content (wt.%)	Cell Size (μm)	Whole-Track Width (μm)	Stable-Region Width (μm)	Whole-Track Depth (μm)	Stable-Region Depth (μm)
0	2.5	55.24	60.48	43.01	47.00
0	5	56.11	61.32	43.71	47.71
0	10	59.95	65.53	46.70	51.07
1.0	2.5	48.84	50.55	46.16	49.24
1.0	5	49.59	51.23	46.89	49.95
1.0	10	53.10	54.81	50.24	53.53

Note: The stable region corresponds to the 0.20–0.40 mm segment along the melt pool length.

**Table 3:** Comparison between the experimental and simulated melt track widths under different laser powers.

Laser Power (W)	Experimental Track Width (μm)	Simulated Track Width (μm)	Relative Deviation (%)
100	45.8	48.5	5.9
200	50.8	54.6	7.5
300	58.6	61.9	5.6
400	63.4	67.1	5.8

**Table 4:** Characteristic Marangoni numbers at different oxygen contents.

O Content (wt.%)	*M*_1_ (N·m^−1^·K^−1^)	*M*_2_ (N·m^−1^·K^−1^)	*Ma*_1_	*Ma*_2_
0.0	+9.941 × 10^−5^	−1.652 × 10^−4^	+32.2	−252.7
0.2	+3.874 × 10^−5^	−1.464 × 10^−4^	+16.5	−190.3
0.4	+9.208 × 10^−5^	−1.383 × 10^−4^	+55.1	−153.0
1.0	+1.471 × 10^−4^	−1.239 × 10^−4^	+113.4	−112.9

**Table 5:** Average track dimensions, vapor-depression depths, and maximum surface temperatures at different oxygen contents.

O Content (wt.%)	Whole-Track Average Width (μm)	Average Width in the Stable Region (μm)	Whole-Track Average Depth (μm)	Average Depth in the Stable Region (μm)	Vapor-Depression Depth (μm)	Maximum Surface Temperature (K)
0.0	56.11	61.32	43.71	47.71	12.33	3741.83
0.2	54.80	57.14	46.23	48.41	14.44	3739.46
0.4	52.20	53.56	46.16	49.29	14.85	3835.18
1.0	49.59	51.23	46.89	49.95	17.04	3895.99

Note: The stable region corresponds to the 0.20–0.40 mm segment along the melt pool length, where the melt pool reaches a quasi-steady state and the influence of scan initiation and termination is minimized.

## Data Availability

The original contributions presented in this study are included in the article. Further inquiries can be directed to the corresponding authors.
